# Electrophysiological evidence for the effectiveness of images versus text in warnings

**DOI:** 10.1038/s41598-023-28230-x

**Published:** 2023-01-23

**Authors:** Wuji Lin, Zhuoyu Li, Xukai Zhang, Yuan Gao, Jingyuan Lin

**Affiliations:** 1 Institute of Brain and Psychological Science, Sichuan Normal University, Chengdu, Sichuan China; 2 Mental Health Education and Counseling Center, Guangdong Teachers College of Foreign Language and Arts, Guangzhou, China; 3 School of Psychology, South China Normal University, Guangzhou, China; 4 Department of Psychology, Faculty of Education and Psychology, University of Jyväskylä, Jyväskylä, Finland; 5 School of Psychology, Shenzhen University, 3688 Nanhai Avenue, Nanshan District, Shenzhen City, China

**Keywords:** Human behaviour, Psychology and behaviour

## Abstract

Warning sign plays an important role in risk avoidance. Many studies have found that images are better warnings than text, while others have revealed flaws of image-only warning signs. To better understand the factors underlying the effectiveness of different types of warning signs (image only, text only, or image and text), this study adopted event-related potential technology to explore the differences at the neurocognitive level using the oddball paradigm and the Go/No-go paradigm. Together, the behavioral and electroencephalogram results showed that text-only warnings had the lowest effectiveness, but there was little difference between the image-only and image-and-text warnings. The differences in the effects of the three warning signs were mainly in the areas of attention and cognitive control, implying differences in the underlying cognitive processes. Therefore, in the design of warning signs, the effects of different design attributes on cognitive processing should be taken into account based on actual needs in order to improve the effectiveness of the signs.

## Introduction

Warning signs are used to alert people about risks in the surrounding environment and help them respond appropriately and immediately by communicating information about the danger, thereby reducing the occurrence of accidents^1^. However, inappropriate or unheeded warnings cause thousands of injuries or deaths each year^2^. Therefore, as a research area of great value to society, the study of warning signs has attracted the attention of a large number of scholars^3,4^.

Previous studies have found that the attributes of warning signs, such as shape^5,6^, border weight^7^, and color^7,8^, affect the responses that the signs elicit. However, the existing research literature shows inconsistent conclusions on the effectiveness of different types of warning signs.

Long and Kearns^9^ found that people are more sensitive to images than to text. In addition, studies on the visibility of warning signs at a distance also found that people saw images first rather than text^10^. These studies showed that images are more effective warnings than text.

However, some studies suggested otherwise. Gonzalez Alam et al.^11^ used the Go/No-go paradigm to explore the influence of different semantics on human inhibitory behavior and found that compared with text, images with the same semantics did not elicit faster responses from the participants. Some studies have explored symbol-only, text-only, and symbol + text stimuli and found that the addition of text can significantly improve the accuracy and shorten the required response time^12^. Lin et al.^13^ compared the warning effects of warning signs with images, text, and images combined with text and found that although the task performance was poorer under the text-only condition, there were no significant differences between the combination condition and the image-only condition.

Although the studies mentioned above explored the differences in warning effects among different types of warning signs, we could hardly understand the reasons for these differences from these studies. An fMRI study by Gonzalez Alam et al.^11^ found that partially overlapping sets of brain regions are involved in the processing of pictures and words. The performance of the target response is related to the interaction between shared control brain regions and brain regions associated with specific inputs or representations. Similar results were also found in a study by Reisch et al.^14^. These results also validate the common semantic system model^15^, which believes that text and pattern information are first processed through different presemantic stages and then enter the semantic processing stage, activating the common semantic processing network.

Since there are differences in the cognitive processing of various types of warning signs, they might also differ in terms of speed and effect. This may ultimately lead to the difference in user performance in interpreting different warning signs. Therefore, exploring the cognitive processing differences of various warning signs is conducive to better design of warning signs and to reducing the occurrence of accidents by using more appropriate warning signs for different scenarios. Reactions to warning signs tend to occur within a short period of time. To study differences in the narrow time window when they occur, event-related potentials (ERPs) have been used. The ERP technique has a high temporal resolution, which therefore enables neural activity to be tracked on a millisecond time scale^16–18^. It enable us to observe the electrophysiological differences between different kinds of warning signs in various time periods, they can help us to know which processing stage are responsible for the differences in behavioral results.

This study investigated attention, inhibition and motion in people’s response to warning signs. The oddball paradigm is one of the most commonly used paradigms to study attention and working memory^19,20^, which requires participants to ignore nontarget stimuli with a high probability of continuous presentation and respond to target stimuli with sudden appearance. Similarly, the Go/No-go paradigm is one of the most commonly used paradigms to study inhibitory processing^21–23^, which requires the participants to respond to the nontarget stimuli with a high probability of continuous presentation and to inhibit the target stimuli with sudden appearance. The two paradigms are useful for understanding the process of execution and inhibition in the response to a suddenly appearing stimulus in an inertial response^13^. The oddball paradigm (Experiment 1) and the Go/No-go paradigm (Experiment 2) were used to investigate the warning effectiveness of the three types of warning signs. We expected that this study would obtain consistent results with the study by Lin et al.^13^. That is, in Experiment 1, we hypothesized that the accuracy for images and combinations would be higher than that for text, while the RT for images would be lower than that for text and combinations. In Experiment 2, we hypothesized that the accuracy for images and combinations would be higher than that for text, while the RT for images would be lower than that for text or combinations.

The main components of time-domain electroencephalogram (EEG) analysis are P2 and N2. As an early positive component, P2 is often associated with attention and is thought to reflect automatic attention to the stimulus^24,25^. In addition, P2 is often considered the early attentional component of threatening stimuli in studies related to warning signs^5,26^. Therefore, the analysis of the P2 component is conducive to better understanding the differences in attentional processing of different warning signs.

N2 is also a common EEG component in cognitive control studies and is thought to reflect top-down inhibitory processing^27^. Some researchers have also found that N2 is related to conflict monitoring^28^. More importantly, research related to warning signs suggests that N2 reflects the advanced stage processing of hazard information^29,30^. Therefore, the analysis of N2 helps to understand the differences in cognitive processing when people decide different warning signs. We hypothesized that the amplitudes of P2 and N2 would be smaller for the text than for the combinations and smaller for the combinations than for the images in both experiments.

In addition to time-domain analysis, time–frequency analysis of EEG data was also performed. A certain frequency of neural oscillation usually represents a certain cognitive process^31^. The theta frequency band is generally believed to be related to cognitive control^32,33^. Other studies have shown that negative or threatening information can trigger a stronger theta oscillation^31,34^. Therefore, theta oscillation analysis can also help to understand the cognitive control and early attention associated with warning signs.

As a much-explored neural oscillation related to motion, mu oscillation is often considered a neural oscillation that reflects movement. More specifically, mu inhibition occurs during movement and can be triggered by motion or motion imagery^35,36^. Through the analysis of mu oscillation, we can understand the difference in the motor response stage when people process different warning signs. We hypothesized that the theta and mu oscillation amplitudes of would be lower for the text than for the combinations and smaller for the combinations than for the images in both experiments.

In brief, two experiments were designed in this study to explore the neural mechanism of warning sign processing by recording and analyzing electrophysiological data.

## Experiment 1: executive processing of different types of warning signs

### Methods

#### Ethics declaration

The procedure in this study was approved by the ethical review board of the School of Psychology, South China Normal University (ID: 2019-4-006) and according to the ethical guidelines of the Helsinki Declaration. All participants took part in this study voluntarily with a written informed online consent form.

#### Participants

The sample size was determined according to power analysis for RTs, Accuracy, EPRs and Time–Frequency. Cohen^37^ defines *fs* of 0.1, 0.25, and 0.4 as small, medium, and large effects, respectively^38^. The *ηp*^*2*^ ranged from 0.154 to 0.381 in previous relevant studies^13^. So, we used a medium effect size of *f* = 0.25 to conduct a power analysis with G*Power 3.1, which suggested that at least 28 participants were required for 80% power to detect the effect given an α level of 0.05. The 80% power is used because it is a commonly accepted for sufficient power^39^. Then, Thirty right-handed participants (mean age 19.8 years) were selected based on previous literature and G*Power’s suggestion. All participants had normal vision or corrected vision and no known mental illness.

#### Materials

**Figure 1:**
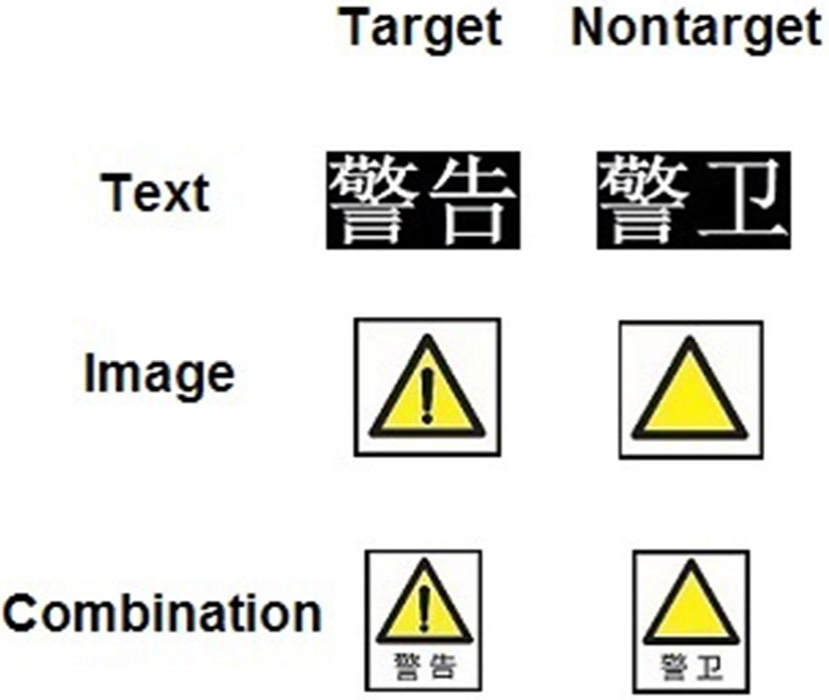
Experimental materials.

The design of images and image-and-text combinations were based on the warning signs in Safety Signs and Guidelines for the Use (Standard No.^40^: black equilateral triangle outlines, black symbols, and yellow background. The sign means "warning" or “to attract attention”. For details, see Fig. 1. Each type of material took up a space of 8 cm^2^ on the screen. There were 60 target stimuli and 180 nontarget stimuli.

The Chinese word in the target stimulus means “warning” (“警告”). The Chinese word in the nontarget stimulus means “guard” (“警卫”).

#### Procedure

The experimental program was compiled with Presentation 0.71 software. The participants completed individual measurements in a soundproofed room. The background of the display monitor was black, and the viewing distance was 80 cm.

The experiment consists of three conditions: text, images, and combinations, with 5-min breaks between conditions. Each stimulus was presented for 500 ms with a random interstimulus interval (ISI) of 1300 ~ 1700 ms. The participants were asked to press the space bar when a target stimulus was presented and withhold pressing when a nontarget stimulus was presented. The execution order of each condition was counterbalanced between participants with a Latin square.

**Figure 2:**
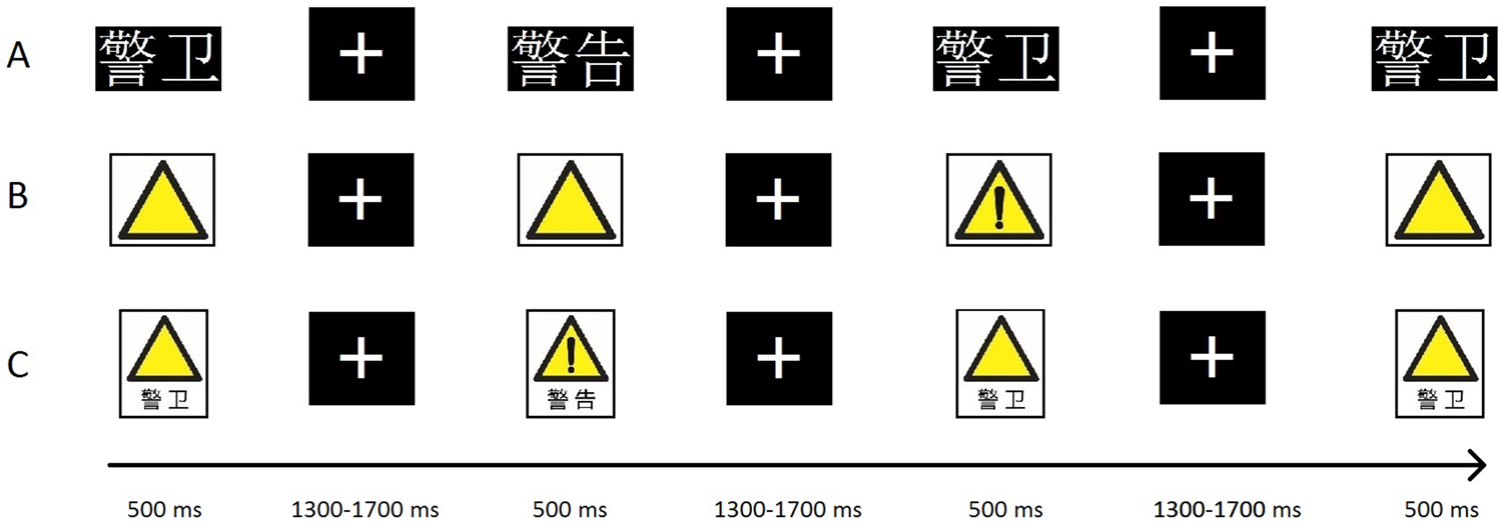
Experimental procedure.

Target and nontarget stimuli are presented in a pseudorandom order with at least one nontarget stimulus separating two target stimuli. See Fig. 2 for the detailed procedure of Experiment 1.

#### Behavioral performance analysis

When a target is presented, the accuracy equals the number of correct keystrokes divided by the total number of targets. The RT of the target is the average RT of the correct responses to the target in the trial. When the nontarget is presented, the accuracy equals the number of times no keystroke response was made divided by the total number of nontargets. Because the participant is not to press the key in response to the nontarget, there is no nontarget RT. SPSS version 24.0 was used for statistical analysis of the data.

#### EEG recording and analysis

Scalp voltage was recorded with a standard international 10–20 system extended NeuroSCAN-64 Ag/AgCl electrode cap using NeuroScan4.5. The analog voltage of the amplifier is 0.05 ~ 100 Hz, and the online sampling rate is 1000 Hz. The ground electrode was located in the middle of the FPz and Fz. The reference electrode was on the top of the head, and the eye electrodes were attached above and below the right eye socket without blocking sight. During the whole experiment, the resistance of all electrodes was less than 10 kΩ.

The Letswave toolbox MATLAB^41^ was used for preprocessing during offline analysis. Invalid trials were excluded. A Butterworth filter was used for 0.1–30 Hz bandpass filtering. After ICA was used to remove electrooculogram (EOG) artifacts, ± 100 μV was used as the standard to exclude other artifacts. Two ICs components were discarded for each participant. The reference was switched to the average voltage of the bilateral mastoid process. For the time domain analysis, segmentations were performed from − 200 ms to 1200 ms, and the baseline correction time was from − 200 ms to stimulus presentation.

To explore the neural activity of participants responding to a sudden target stimulus in the inertial response, the difference wave between target and nontarget was used for the analysis of brain wave amplitudes, and the difference between target and nontarget was also used for the analysis of neural oscillations. To explore the cognitive processing speed of the target, the latency of the target was calculated.

P2: According to previous literature^24,29^ combined with the results of the present study, the average amplitude of six electrode points (F3, Fz, F4, C3, Cz, and C4) with a time window of 155–195 ms was selected as the mean amplitude of P2, and the latency was defined as the time during the presence of stimulus to the peak point of amplitude within the time window.

N2: According to previous literature^29,42^ combined with the results of the present study, the average amplitude of six electrode points (F3, Fz, F4, C3, Cz, and C4) with a time window of 200–350 ms was selected as the mean amplitude of N2, and the latency was defined as the time during the presence of stimulus to the peak point of amplitude within the time window.

For time–frequency analysis, the Morlet wavelet transform (CMOR1-1.5) was used to decompose the segmented EEG signals. The decomposition frequency was 1–30 Hz, and the step of frequency was 1 Hz. To avoid the influence of wavelet transform on the edge, the EEG signals from 1000 ms before stimulation and 1200 ms after stimulation were extracted, and the baseline correction was performed at − 750 ~ − 250 ms. In the baseline correction, positive values represent event-related synchronization, and negative values represent event-related desynchronization. Baseline correction is carried out according to the following formula:

$${\text{ERS}}/{\text{ERD }} = \, \left[ {{\text{AP }}\left( {\text{t}} \right) \, - {\text{ AP baseline}}} \right]/{\text{AP baseline }}\left( {\text{AP means average power in the time window}} \right).$$

Theta: According to previous literature^31^ and combined with the results of the present study, 4–8 Hz neural oscillations at 100–500 ms of Cz electrode were selected as the regions of interest (ROIs). Then, we performed time–frequency decomposition in three steps. First, we performed a wavelet decomposition analysis to obtain six theta oscillation outcomes of our experimental conditions. Second, we calculated the theta oscillation difference by subtracting the theta oscillation outcomes of target and non-target conditions (target minus non-target). Third, we compared the theta oscillation difference among text, image, and combination conditions using ANOVA.

Mu: According to previous literature^43,44^ and combined with the results of this study, 8–13 Hz neural oscillations at 250–600 ms of C3 and C4 electrodes were selected as the ROIs. In line with theta oscillation, we performed time–frequency decomposition in three steps. First, we performed a wavelet decomposition analysis to obtain six Mu oscillation outcomes of our experimental conditions. Second, we calculated the Mu oscillation difference by subtracting the theta oscillation outcomes of target and non-target conditions (target minus non-target). Third, we compared the Mu oscillation difference among text, image, and combination conditions using ANOVA.

#### Statistical analysis

The accuracy of the target and the nontarget responses did not fulfill the assumptions of normality required by ANOVA and could not be transformed to a normal distribution. Accordingly, the Friedman test was performed on the accuracy of the target and the nontarget responses. The response time of the target and P2 latency were transformed (reciprocal transformation) to better approximate a normal distribution. Other results fulfilled the assumptions of normality. One-factor, three-level repeated-measures ANOVA was performed on the response time of the target, P2 difference wave, P2 latency, N2 difference wave, N2 latency, theta difference oscillations, and mu difference oscillations. Greenhouse‒Geisser adjustments were applied, as needed, to correct for violations of sphericity. Follow-up analyses of the simple effect via Bonferroni's adjustment were separately executed for each condition.

### Results

#### Behavioral results

**Figure 3:**
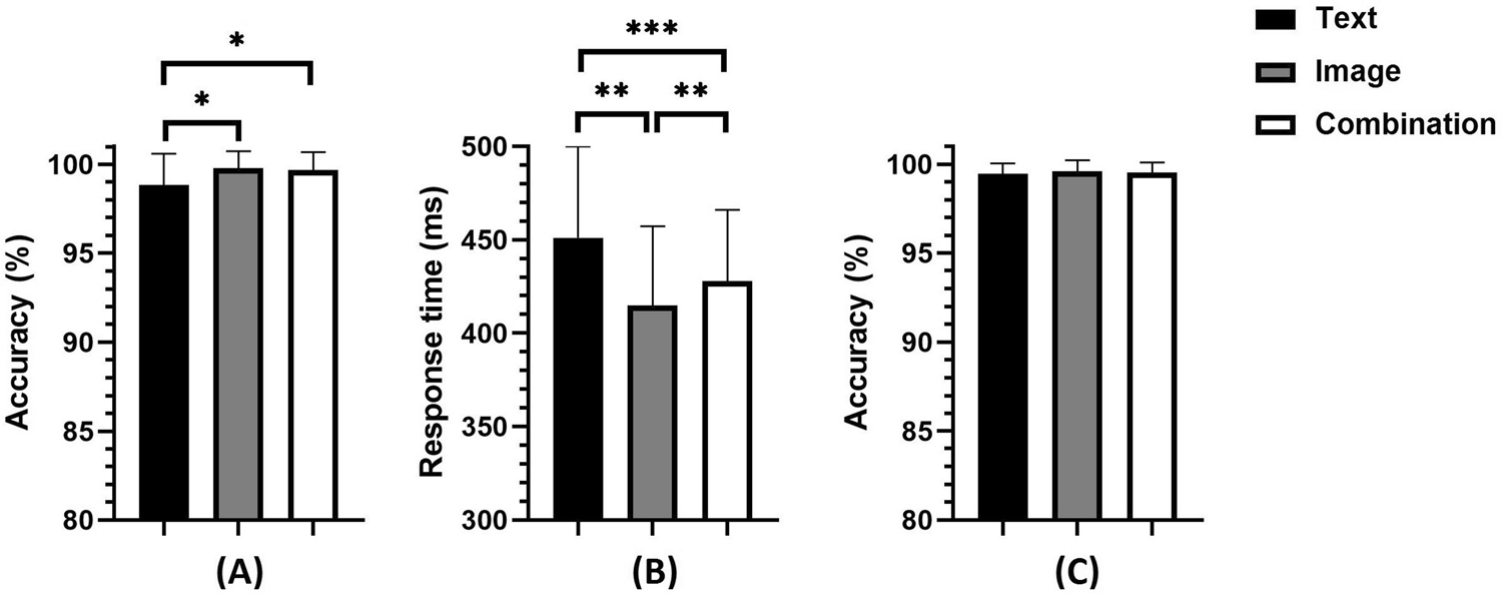
Accuracy and response time in the oddball task: (**A**) accuracy for the target; (**B**) response time of the target; (**C**) accuracy for the nontarget. *p < 0.05; **p < 0.01; ***p < 0.001.

First, the Friedman test with Dunn’s post-hoc test was performed on the accuracy of the target responses (images vs. text vs. combination), and the results showed a significant main effect (*χ*^2^ = 21.784, *p* < 0.001). Post-hoc test revealed higher accuracy for the images than for the text (*p* < 0.05), and for the combination than for the text (*p* < 0.05), there were no significant differences between images and combinations (*p* = 0.747). See Fig. 3A and Table 1.

**Table 1:** Accuracy and response time in the oddball task (SD).

	Text	Image	Combination
Accuracy for target (%)	98.83 (1.76)	99.78 (0.95)	99.67 (1.02)
Response time for target (ms)	451.14 (48.86)	414.78 (42.52)	427.87 (38.24)
Accuracy for nontarget (%)	99.46 (0.59)	99.59 (0.63)	99.52 (0.58)

After that, repeated-measures ANOVA was performed on the response time of the target, and the results showed that the main effect was significant (*F*(2,58) = 22.687, *p* < 0.001, *ηp*^2^ = 0.439). The post hoc test found a shorter reaction time for the images than for the text (*p* < 0.001) and for the images than for the combinations (*p* < 0.01), and the reaction time of the combinations was less than that of the text (*p* < 0.01). See Fig. 3B and Table 1.

A Friedman test of nontarget accuracy was also conducted. The main effect was not significant (*χ*^2^ = 2.164, *p* = 0.339). See Fig. 3C and Table 1.

#### Electrophysiological results

**Figure 4:**
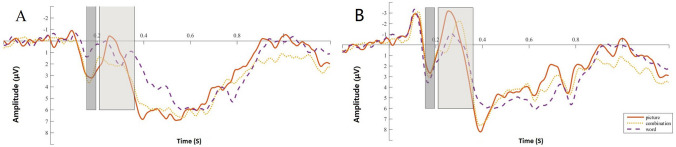
Difference wave and latency in the oddball task: (**A**) difference wave between target and nontarget; (**B**) mean amplitude of target. Shaded areas are the time range of the P2 component (155–195 ms) and N2 component (200–350 ms). All of the images of ERPs are the result averaged by six electrodes (FC3, FCz, FC4, C3, Cz, and C4).

First, repeated-measures ANOVA was performed on the P2 difference wave (images vs. text vs. combination), and the main effect was significant (*F*(2,58) = 14.929, *p* < 0.001, *ηp*^2^ = 0.340). A post hoc test showed a smaller amplitude for the text than for the images (*p* < 0.001) and a smaller amplitude for the text than for the combinations (*p* < 0.001). There were no significant differences between the combinations and the images (*p* = 0.922). See Fig. 4A. Repeated-measures ANOVA was also performed for the P2 latency of the target, and the main effect was not significant (*F*(1.484,43.046) = 0.860,* p* = 0.400, *ηp*^2^ = 0.029). See Fig. 4B.

Repeated-measures ANOVA was also conducted for the N2 difference wave, and the main effect was significant (*F*(1.627,47.180) = 3.591, *p* < 0.05, *ηp*^2^ = 0.110). A post hoc test showed no significant differences between the text and the images (*p* = 0.574). The amplitude for the text was smaller than for the combinations (*p* < 0.01). The differences between the combinations and the images were marginally significant (*p* = 0.082). See Fig. 4A. Repeated-measures ANOVA was also performed for the N2 latency of the target, and the main effect reached marginal significance (*F*(2,58) = 2.813, *p* = 0.068, *ηp*^2^ = 0.088). A post hoc test showed no significant differences between the text and the images (*p* = 0.526). There were no significant differences between the text and the combinations (*p* = 0.099). The latency of the combinations was larger than that of the images (*p* = 0.050). See Fig. 4B.

**Figure 5:**
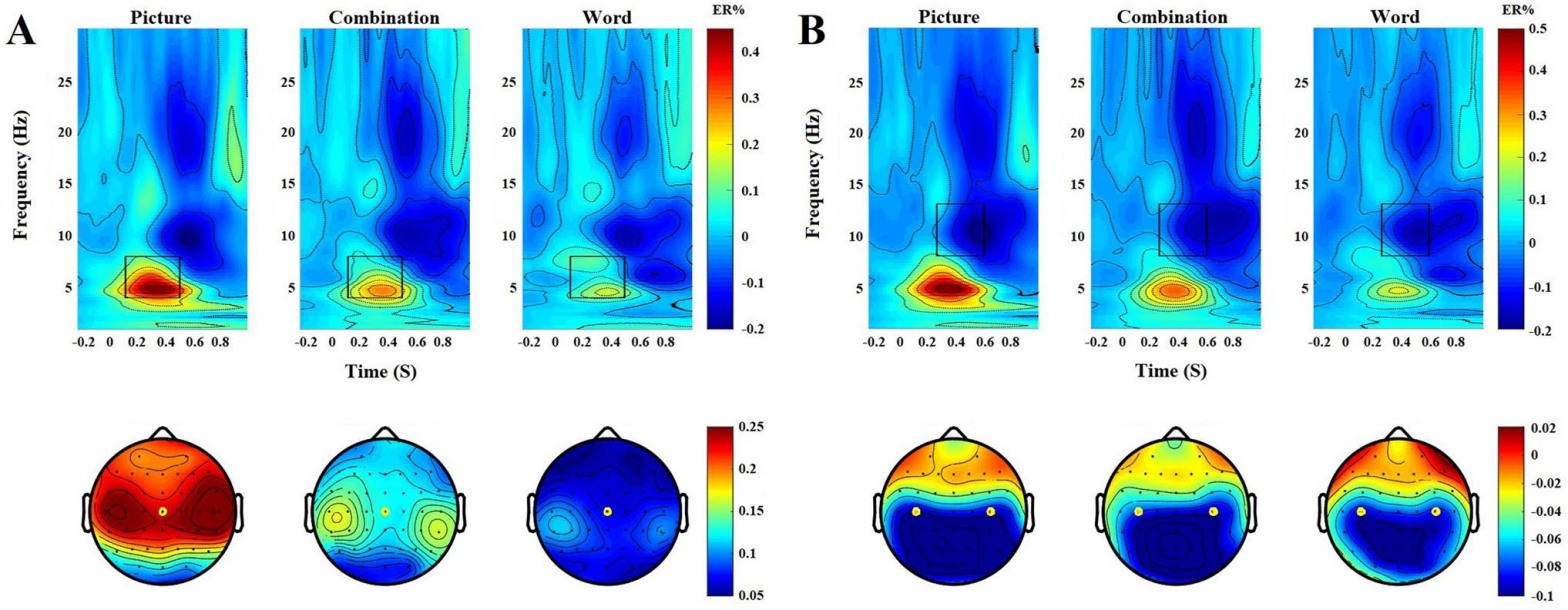
Difference oscillation in the oddball task: (**A**) time–frequency of theta band; (**B**) time–frequency of mu band. Rectangular areas are the range of time and frequency of the theta band and the mu band (theta: 4–8 Hz neural oscillation at 100–500 ms; mu: 8–13 Hz neural oscillation at 250–600 ms).

Repeated-measures ANOVA was performed for the theta difference oscillations, and the main effect was significant (*F*(2,58) = 15.024, *p* < 0.001, *ηp*^2^ = 0.341). A post hoc test showed that the oscillation amplitude of the text was less than that of the images (*p* < 0.001). The differences between the text and the combinations were significant (*p* < 0.05). The oscillation amplitude of the combinations was less than that of the images (*p* < 0.01). See Fig. 5A.

Repeated-measures ANOVA on mu difference oscillations showed that the main effect was not significant (*F*(2,58) = 1.417, *p* = 0.251, *ηp*^2^ = 0.047). See Fig. 5B.

### Discussion

Consistent with previous research results^13^, the warning effect of text was the worst, indicated by lower accuracy and longer reaction time. The warning effect of signs with images was the best. There were differences in reaction time between the combinations and the images but not in accuracy. Furthermore, electrophysiological results showed significant differences between different warning signs. The images had the largest amplitude, shortest latency, and largest neuronal oscillation amplitude. The text was the opposite of the images. The combinations were similar to the images, but only some of the components showed differences.

In this experiment, the participants were required to press the key when presented with the targets that suddenly appeared. Both the image and combination conditions had high accuracy, while the accuracy of the text condition was low. These results were consistent with the performance of P2 amplitude in electrophysiological data. P2 reflects the early attention to stimuli^5,24,26^. Consistent results showed that pictures can attract more attention and elicit more correct responses from the participants. There were no significant differences in the ability of combinations and images to attract early attention, indicating that combinations can also attract strong attention. However, the attention effect caused by text is weaker than that caused by combinations and images.

In addition, theta oscillation results were similar to the reaction time. Cooper et al.^45^ found that in cognitive control, theta oscillation results are closely related to reaction time. Intracranial records of nonhuman primates show that theta oscillations originate from the mPFC, which is widely believed to play a key role in goal-oriented behavioral control^46–49^. In this study, the differences in theta oscillation results among different types of warning signs and their correlation with reaction time may be because under the image condition, the participants can distinguish the target from the nontarget with a high degree of certainty and press the key quickly. This allowed participants to have stronger cognitive control when making decisions, accompanied by shorter reaction times. In contrast, it was more difficult for the participants to distinguish the target from the nontarget under the text condition, which resulted in weakened cognitive control with longer reaction times.

Smaller differences in N2 components and mu oscillations indicated that there were no significant differences in inhibitory processing and motor responses among the three types of warning signs in oddball tasks requiring responses to targets with a low probability of appearance. The differences among the three types of warning signs are more related to attention and cognitive control.

## Experiment 2: inhibition in response to different types of warning signs

### Method

#### Participants

Consistent with Experiment 1, 30 participants were selected. The average age was 20.3 years. The participants were right-handed, had normal vision or corrected vision and had no known mental illness.

#### Material

Same as Experiment 1.

#### Procedure

Participants were required to press the space bar when presented with a nontarget stimulus and withhold pressing when a target stimulus was presented. The rest are the same as in Experiment 1.

#### Behavioral performance analysis

When the nontarget was presented, the accuracy equals the number of correct keystrokes divided by the total number of nontargets. The RT of the nontarget is the average RT of the correct responses to the nontarget in the trial. When the target was presented, the accuracy equals the number of times keystrokes were withheld divided by the total number of targets. Because the participant does not press the key in response to the target, there is no target RT. SPSS version 24.0 was used for statistical analysis of the data.

#### EEG recording and analysis

Same as Experiment 1.

#### Statistical analysis

The response times to the nontarget stimuli fulfilled the assumptions of normality. The N2 latency was transformed (reciprocal transformation). Others same as Experiment 1.

### Results

#### Behavioral results

**Figure 6:**
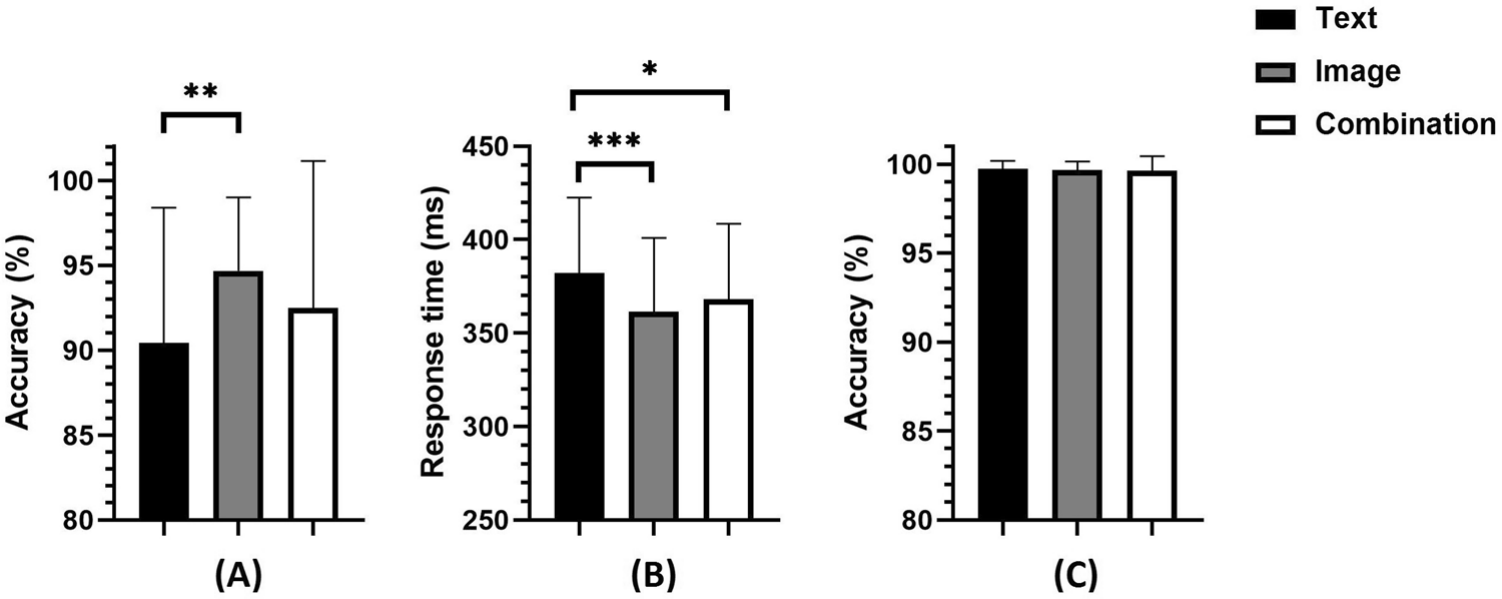
Accuracy and response time in the Go/No-go task: (**A**) accuracy for target; (**B**) response time of target; (**C**) accuracy for nontarget. *p < 0.05; **p < 0.01; ***p < 0.001.

First, the Friedman test with Dunn’s post-hoc test was performed on the accuracy of the target (images vs. text vs. combinations), and the results showed a marginally significant main effect (*χ*^2^ = 8.222, *p* < 0.05). A Post-hoc test revealed higher accuracy for the images than for the text (*p* < 0.01), the differences between the images and combinations were not significant (*p* = 0.121), and there were no significant differences between the combinations and the text (*p* = 0.245). See Fig. 6A and Table 2.

**Table 2:** Accuracy and response time in the Go/No-go task (SD).

	Text	Image	Combination
Accuracy for target (%)	90.43 (7.96)	94.67 (4.34)	92.50 (8.64)
Response time for target (ms)	382.03 (40.48)	361.65 (39.24)	368.21 (40.36)
Accuracy for nontarget (%)	99.73 (0.45)	99.67 (0.48)	99.63 (0.81)

After that, repeated-measures ANOVA was performed on the response time to the nontarget stimuli, and the results showed that the main effect was significant (*F*(2,58) = 8.796, *p* < 0.001, *ηp*^2^ = 0.233). The post hoc test found shorter reaction times for the images than for the text (*p* < 0.001) and for the combinations than for the text (*p* < 0.05), and there were no significant differences between the combinations and the images (*p* = 0.152). See Fig. 6B and Table 2.

A Friedman test for nontarget accuracy was also conducted and yielded a nonsignificant main effect (*χ*^2^ = 0.533, *p* = 0.766). See Fig. 6C and Table 2.

#### Electrophysiological results

**Figure 7:**
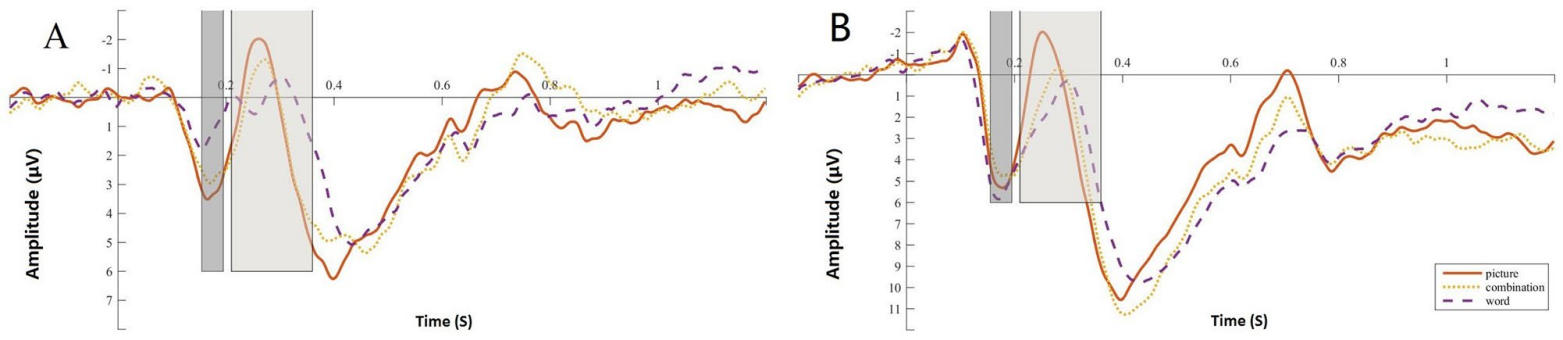
Difference wave and latency in the Go/No-go task: (**A**) difference wave between target and nontarget; (**B**) mean amplitude of target.

First, repeated-measures ANOVA was performed for P2 different waves (images vs. text vs. combination), and the main effect was significant (*F*(2,58) = 12.673, *p* < 0.001, *ηp*^2^ = 0.304). A post hoc test showed a smaller amplitude for the text than for the images (*p* < 0.001) and for the text than for the combinations (*p* < 0.01). There were no significant differences between the combinations and the images (*p* = 0.191). See Fig. 7A. Repeated-measures ANOVA was also performed for the P2 latency of the target, and the main effect was not significant (*F*(1.497,46.422) = 2.198, *p* = 0.120, *ηp*^2^ = 0.066). See Fig. 7B.

Repeated-measures ANOVA was also performed for N2 different wave and the main effect was not significant, (F(2,58) = 1.999, *p* = 0.145, *ηp*^2^ = 0.064). See Fig. 7A. Repeated-measures ANOVA was also performed for the N2 latency of the target, and the main effect was significant (*F*(2,58) = 6.695, *p* < 0.01, *ηp*^2^ = 0.188). A post hoc test showed a larger latency for the text than for the images (*p* < 0.01) and for the combinations than for the images (*p* < 0.05). There were no significant differences between the text and the combinations (*p* = 0.603). See Fig. 7B.

**Figure 8:**
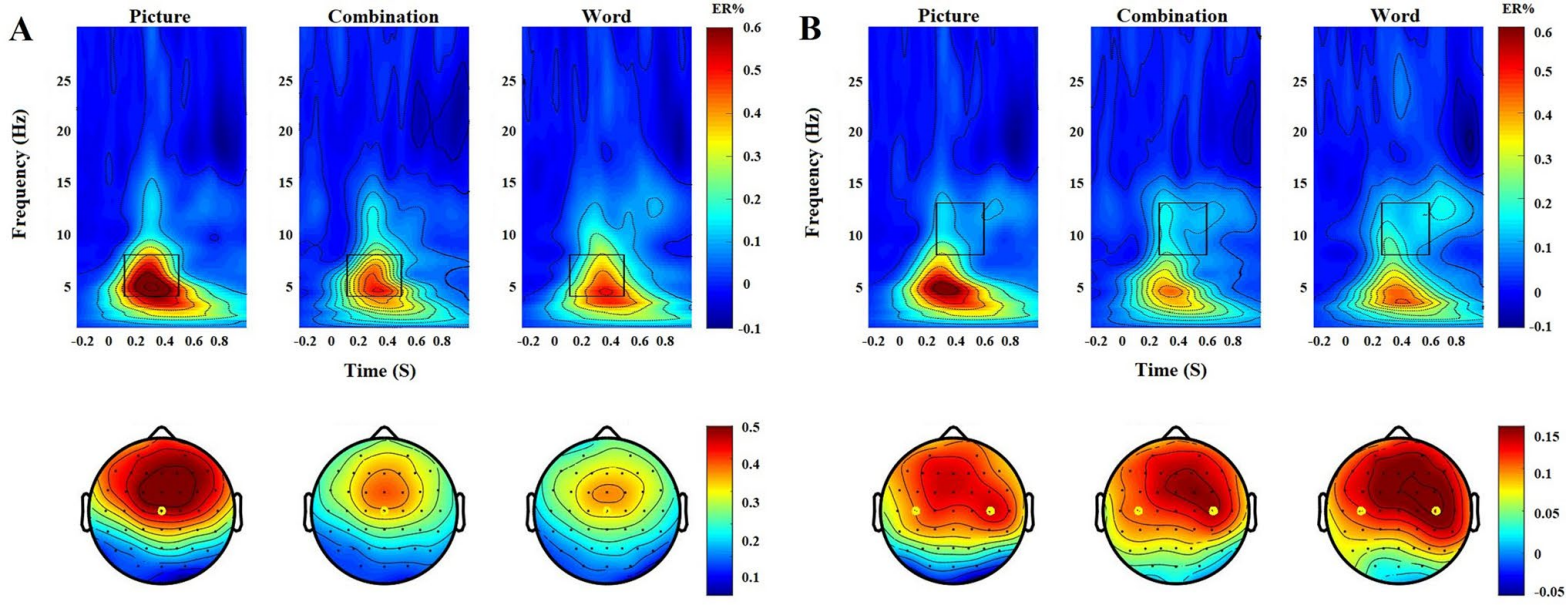
Difference oscillation in the Go/No-go task: (**A**) time–frequency distribution of theta-band activity; (**B**) time–frequency distribution of mu-band activity.

Repeated-measures ANOVA was performed for the theta difference oscillations, and the main effect was significant (*F*(2,58) = 10.474, *p* < 0.001, *ηp*^2^ = 0.265). A post hoc test showed that the oscillation amplitude of the text was smaller than that of the images (*p* < 0.001). There were no significant differences between the text and the combinations (*p* = 0.512). The oscillation amplitude of the combinations was smaller than that of the images (*p* < 0.01). See Fig. 8A.

Repeated-measures ANOVA on mu difference oscillations showed that the main effect was not significant (*F*(1.550,44.948) = 0.598, *p* = 0.553, *ηp*^2^ = 0.020). See Fig. 8B.

### Discussion

In this experiment, the images also had the highest accuracy among the three, while there were no significant differences in accuracy between the combinations and the text. The behavior results were consistent with the theta oscillations in the electrophysiological data. As mentioned above, theta oscillations are usually associated with cognitive control^32,33^. The results showed that in inhibitory processing, the images could also enhance the cognitive control ability of the participants when making decisions and elicit a higher accuracy in the behavioral results. However, the combinations did not have a positive effect on cognitive control as the images did in Experiment 1, which may be because inhibition of inertial response requires stronger cognitive control ability. The combinations in the Go/No-go task did not have an effect on cognitive control as strongly as the images did. Therefore, the amplitude of theta oscillations for the combinations is lower than that for the images, accompanied by lower accuracy.

In the Go/No-go task, the participants pressed the key to the nontarget and refrained from pressing to the target. To be successful, participants must identify the strategy that optimally balances the following two goals: respond as quickly and as accurately as possible to the nontarget and withhold the response to the target as effectively as possible^27^. Although there were no response time data for the target, the cognitive processing differences of the participants when judging different warning signs could be understood by analyzing the accuracy and time of responses to the nontarget^13^. This study found that the results concerning the nontarget response time were consistent with those of the amplitude of the P2 different wave. Ma et al.^50^ proposed the "hazard perception two-stage" (HPTS) model, that is, people need to undergo early perception and late semantic processing to process risk information. The study of Bian et al.^29^ further proves that the processing of warning signs includes the stage of rapid recognition of danger information represented by P2 and the stage of conscious integration of danger information represented by N2 in working memory. Therefore, the results of P2 amplitude in this study may be because, compared with text, images and combinations can evoke a higher level of danger perception. There were no significant differences between images and combinations in hazard perception.

In this experiment, we also found no differences in mu oscillations between different warning signs. This means that in the inhibition processing task, there were no differences in the motor response processing of the three warning signs.

## General discussion

The behavioral results of this study suggested the worst warning effect with text and the best effect with images. The combinations yielded better performance than the text and worse than the images in terms of accuracy for Experiment 1 and in terms of response time for Experiment 2. However, there were no significant differences between the warning effect of the text and the images. In terms of neurocognitive results, the differences in EEG components were mainly manifested in the P2 component and theta oscillation.

P2 differences between the three warning signs were consistent within the two tasks. P2 mainly reflected early attention processing, and the differences were mainly caused by the attributes of the stimulus^29,51^. Some researchers have found that the range of ERP components identified in the two paradigms were largely equivalent. This finding indicated that similar neurocognitive processes are required in each task^52^. Various aspects of cognitive control, such as goal maintenance and attentional regulation, are required in both tasks^11^. Whether in execution processing tasks or inhibition processing tasks, the participants' early attentional processing of the target was similar, but the subsequent processing showed differences. The text had the smallest P2 amplitude, while there were no differences between the images and the combinations. The two experiments consistently demonstrated that text was less effective in attracting early attention. Images and combinations enabled the participants to decide faster, and there were no significant differences between the two. The poor effectiveness of text in attracting early attention was reflected in the behavioral performance: 1 When required to respond to suddenly appearing targets (Experiment 1, participants were prone to make wrong responses,2 When required to refrain from responding to the suddenly appearing target (Experiment 2, the participants were more cautious in deciding the targets and nontargets, which led to longer nontarget response time.

Although there were significant differences between the three types of warning signs, these differences were not consistent between the two tasks and were mainly reflected in the combinations. Some researchers believe that the core domain of cognitive function in the oddball task is selective attention. The core domain of cognitive function in the Go/No-go task is response inhibition^53,54^. In Experiment 1, theta oscillations were the smallest for the text and the largest for the images, while the combinations were somewhere in between. In Experiment 2, the performances for the text and the images were the same as in Experiment 1, but the differences between the combinations and the text were not significant. In inhibitory processing, participants were required to press the key to the nontarget and refrain from pressing to the target. Theta oscillations primarily reflect cognitive control^32,33,45^. The theta oscillations of both combinations and text were smaller and accompanied by lower accuracy than those of the images. This may be because it was not easy to distinguish the target from the nontarget in the rapid presentation process, and it was difficult for participants to make decisions in the task. Therefore, when processing nontargets, more cognitive resources were needed to make keystroke responses after correct discrimination. However, in the selective attention process, the participants needed to ignore the nontarget. Theta oscillations reflect more cognitive control of the target. Because the combinations can attract more early attention than the text, participants could have better cognitive control and make better decisions (higher accuracy and shorter response time). However, the compound stimuli are different from the image alone because there are two stimuli to be processed. They may not be integrated as a whole. More information leads to greater cognitive load. Therefore, it has a longer reaction time relative to the image.

No significant differences were observed in mu oscillations in the two experiments, indicating that the differences in behavioral results of different types of warning signs were mainly caused by cognitive processing rather than behavioral reaction. There were no significant differences in the reaction stage after target or nontarget processing was completed.

### Limitations and prospects

First, although the present study found behavioral and electrophysiological differences between different types of warning signs in the two tasks, they were designed only to explore the differences in cognitive processing, and the roughly simulated emergency situations were quite different from those in the real world. More ecological methods can be used for further research, such as driving simulators, VR technology, and experiments in real scenes. Second, the tasks used in this study were relatively simple, and further research could employ different experimental paradigms to explore different aspects of the question. Third, emergencies are often accompanied by a variety of conditions. In the future, we can explore the differences in cognitive processing under different conditions, such as fatigue, sleep deprivation, and interference. Fourth, although EEG results have a high temporal resolution, they have the shortcoming of low spatial resolution, and the conclusions drawn solely from EEG results are not completely reliable. The results can be verified using magnetic resonance imaging, near-infrared detection, and other technologies in the future. Fifth, there was a ceiling effect in behavioral results. The results can be verified using more sensitive tasks in the future. Sixth, power analysis is performed based on the effect size of a single study. The results can be verified using larger sample size in the future.

This study explored behavioral and neurocognitive differences among the three types of warning signs. The results showed that images had the best behavioral performance because they could induce strong early attention and cognitive control but were not related to motor responses represented by mu oscillations. Text worked worst, and the combinations were somewhere in between.

Therefore, easy-to-understand images should be used as warning signs in scenarios requiring an emergency response. When the situation is complex and does not require a quick response, text or combinations can be used. In addition, more attention-attracting elements should be included in the design to improve the effectiveness of early attention and cognitive control.

## Supplementary Information

Supplementary Information.

## Data Availability

The datasets used and/or analysed during the current study available from the corresponding author on reasonable request.
